# Characterization of Bile Salt Hydrolase from *Lactobacillus gasseri* FR4 and Demonstration of Its Substrate Specificity and Inhibitory Mechanism Using Molecular Docking Analysis

**DOI:** 10.3389/fmicb.2017.01004

**Published:** 2017-05-31

**Authors:** Rizwana Parveen Rani, Marimuthu Anandharaj, Abraham David Ravindran

**Affiliations:** ^1^Department of Biology, The Gandhigram Rural Institute – Deemed UniversityGandhigram, India; ^2^Biodiversity Research Center, Academia SinicaTaipei, Taiwan

**Keywords:** bile salt hydrolase, *Lactobacillus gasseri* FR4, antibiotics used as growth promoters, homology modeling, docking analysis

## Abstract

Probiotic bacteria are beneficial to the health of poultry animals, thus are used as alternative candidates for antibiotics used as growth promoters (AGPs). However, they also reduce the body weight gain due to innate bile salt hydrolase (BSH) activity. Hence, the addition of a suitable BSH inhibitor along with the probiotic feed can decrease the BSH activity. In this study, a BSH gene (981 bp) encoding 326-amino acids was identified from the genome of *Lactobacillus gasseri* FR4 (*Lg*BSH). The *Lg*BSH-encoding gene was cloned and purified using an *Escherichia coli* BL21 (DE3) expression system, and its molecular weight (37 kDa) was confirmed by SDS–PAGE and a Western blot analysis. *Lg*BSH exhibited greater hydrolysis toward glyco-conjugated bile salts compared to tauro-conjugated bile salts. *Lg*BSH displayed optimal activity at 52°C at a pH of 5.5, and activity was further increased by several reducing agents (DTT), surfactants (Triton X-100 and Tween 80), and organic solvents (isopropanol, butanol, and acetone). Riboflavin and penicillin V, respectively, inhibited *Lg*BSH activity by 98.31 and 97.84%. A homology model of *Lg*BSH was predicted using *Ef*BSH (4WL3) as a template. Molecular docking analysis revealed that the glycocholic acid had lowest binding energy of -8.46 kcal/mol; on the other hand, inhibitors, i.e., riboflavin and penicillin V, had relatively higher binding energies of -6.25 and -7.38 kcal/mol, respectively. Our results suggest that *L. gasseri* FR4 along with riboflavin might be a potential alternative to AGPs for poultry animals.

## Introduction

In recent years, probiotics have been considered as alternative candidates for antibiotics used as growth promoters (AGPs). Basically, AGPs are group of antibiotics (i.e., bambermycin, lincomycin, tylosin, etc.) used in animal feed at sub-therapeutic levels to improve the growth performance and average body mass gain of food animals (Wang et al., 2012). However, negative impacts of AGPs include the emergence of antibiotic-resistant bacteria which may be transmitted to humans and cause food safety threats and public health issues (Marshall and Levy, 2011; Lin, 2014). European Union member countries have banned use of all AGPs for food animals (Marshall and Levy, 2011). However, banning AGPs has severely affected the health and productivity of poultry animals in several countries (Casewell et al., 2003). Hence, the use of probiotics has emerged as an alternative for AGPs and several health-promoting effects were observed (Lin, 2011). Although probiotic feed supplements improve the growth performance of poultry animals, substantial losses or no significant increases in body weight were observed in probiotic (especially *Lactobacillus* strains) treated animals (Million et al., 2012; Angelakis et al., 2013). This is due to the production of higher amounts of bile salt hydrolase (BSH) enzymes. Generally, BSH activity is considered as a functional probiotic biomarker due to its health-protective effects (i.e., cholesterol reduction, bile tolerance, antimicrobial activity, etc.) (Miremadi et al., 2014). BSH activities may contribute to microbial bile resistance, colonization of the gastrointestinal tract (GIT), host metabolism and energy harvest (Begley et al., 2006; Lin et al., 2014). Probiotic microorganisms have the ability to transform bile salts to a great extent through a bile salt deconjugation mechanism. Bile salts are synthesized from cholesterol, conjugated with glycine or taurine in the liver, stored in the gall bladder and secreted into the small intestine (Kim and Lee, 2005). Bile salts play a significant role in lipid digestion and act as a biological detergent (Hofmann and Eckmann, 2006). The BSH enzyme has the ability to hydrolyze conjugated bile salts into a deconjugated form and release free amino acids. Deconjugated bile salts are much less soluble, hence are not absorbed by intestinal cells and are excreted in feces. This mechanism results in higher utilization of cholesterol for the *de novo* synthesis of bile acids, thereby lowering the serum cholesterol levels (Begley et al., 2006).

To be used as an alternative for AGPs, BSH activities of probiotic bacteria should be inhibited by a specific inhibitor, which could be supplemented along with the probiotic feed. This will dramatically decrease the BSH activity and increase fat deposition in poultry animals (Joyce and Gahan, 2014). Recently several researchers have identified potential BSH inhibitors including gossypetin, caffeic acid phenethyl ester (CAPE), epicatechin monogallate, riboflavin and demonstrated the inhibition of BSH enzyme activity (Lin et al., 2014; Smith et al., 2014). However, BSHs of different microorganism have different protein structures and substrate specificities. Identification of potential BSH inhibitors relies on the availability of defined crystal structures of BSH enzymes (Smith et al., 2014). Hence modern computational strategies, such as homology modeling and molecular docking studies can be used to identify safe, potent and cost-effective BSH inhibitors using *in silico* analysis. The identified novel BSH inhibitors can then be used alongside BSH-positive probiotic microorganisms to decrease host fat digestion in food animals and in turn enhance the profitability of feed-additive industries (Knarreborg et al., 2004; Wang et al., 2012; Lin, 2014). In our previous study, a probiotic *Lactobacillus gasseri* FR4 from the GIT of free-range chickens (*Gallus gallus* subsp. *domesticus*) was isolated and efficiently hydrolyzed taurodeoxycholate (TDC) to deoxycholate on de Man Rogosa Sharpe (MRS)-TDC agar plate (Parveen Rani et al., 2016). We hypothesize that this hydrolysis activity is due to the production of a BSH enzyme. To use this probiotic bacteria as an alternative for AGPs, this BSH enzyme activity should be inhibited. Therefore, we sought to find a potential candidate to inhibit BSH activity.

In this study, we have identified a gene responsible for BSH activity of the probiotic *L. gasseri* FR4, which was cloned and the enzyme overexpressed, purified and characterized. Several compounds were screened to identify a potential BSH inhibitors and riboflavin exhibited higher percentage of inhibition. The protein structure of newly identified BSH enzyme was modeled using homology modeling. Molecular docking analysis was performed to identify the substrate specificity and inhibitory mechanism of identified inhibitors.

## Materials and Methods

### Bacterial Strains and Plasmids

The probiotic *L. gasseri* FR4 was previously isolated from the GIT of free-range chickens (Parveen Rani et al., 2016). The strain was routinely subcultured in MRS media at 37°C under anaerobic conditions. *Escherichia coli* DH5α and BL21 (DE3) strains were, respectively, used for the cloning and expression of the BSH enzyme. The *E. coli* expression vector, pET21b (+), was used for expression of His-tagged (6x) recombinant BSH. The cloning and expression hosts were maintained in Luria-Bertani (LB) broth supplemented with ampicillin (100 μg/ml) at 37°C under aerobic conditions.

### Identification of the BSH Gene

To identify the putative BSH gene, we mined the available whole-genome sequence of *L. gasseri* ATCC 33323 = JCM 1131 (NC_008530.1) using the Basic Local Alignment Search Tool (BLAST) algorithm from the National Center for Biotechnology Information (NCBI)^1^. The putative BSH gene was aligned with other bacterial BSHs using the Clustal omega multiple sequence alignment tool^2^, and the secondary structure was predicted using ESpript 3.0^3^ (Robert and Gouet, 2014). The phylogenetic relationship of the identified putative BSH of *L. gasseri* FR4 was confirmed by an unweighted pair group method with arithmetic mean (UPGMA) phylogenetic tree using MEGA6 software (Tamura et al., 2013).

### Genomic DNA Extraction and Cloning of the BSH Gene

Genomic DNA of *L. gasseri* FR4 was extracted using a DNeasy Blood & Tissue Kits (Qiagen, Germany) according to the manufacturer’s instructions. The putative BSH gene (975 bp) was amplified from the purified genomic DNA using *Lg*BSH-F (5′-gcgGGATCCTGTACCTCAATTATTT-3′) and *Lg*BSH-R (5′-gagCTCGAGATTTTGATAGTTAATATG-3′) primer pairs, respectively, flanked with *Bam*HI and *Xho*I (the underlined sequences) (Supplementary Figure S1). The amplified polymerase chain reaction (PCR) product was purified using a QIAquick PCR purification kit (Qiagen, Germany). pET21b (+) vector DNA and the amplified *Lg*BSH-encoding gene were digested with *Bam*HI and *Xho*I (New England Biolabs), resolved in a 1% agarose gel, extracted from the gel and ligated using T4 DNA ligase (ThermoFisher Scientific). The resulting plasmid was transformed into *E. coli* DH5α-competent cells and plated on LB agar plates with ampicillin (100 μg/ml). The plasmid was extracted and sequenced using T7-F and T7-R universal primers and no mutations in the coding sequence of *Lg*BSH-encoding gene were detected (Supplementary Figure S2).

### Expression and Purification of Recombinant *Lg*BSH

To express the recombinant *Lg*BSH, the pET21b-BSH plasmid was purified from *E. coli* DH5α and transformed into the expression host *E. coli* BL21 (DE3). Protein expression and purification was performed as described in Kumar et al. (2013). Briefly, *E. coli* BL21 (DE3) cells harboring the pET21b-BSH plasmid were cultured overnight and cells with an optical density (OD) of 0.15 were inoculated into 50 ml of LB broth with ampicillin. When the culture reached 0.6 OD, 500 μl of 100 mM isopropyl-β-d-thiogalactopyranoside (IPTG) was added and incubated for 3 h to allow protein expression. Then, centrifugation was performed at 8000 rpm for 10 min at 4°C. Cells were resuspended in lysis buffer containing 50 mM Tris-HCl (pH 7.5), 100 mM NaCl, 1 mM dithiothreitol (DTT), and 1x SIGMAFAST^TM^ protease inhibitor cocktail (Sigma–Aldrich, St. Louis, MO, United States). Then, sonication was performed at six cycles of 20 s on/off with a 60% amplitude and 50% duty cycle on ice using a 3-mm probe (Sonics Vibracell-VCX130, United States). Sonicated cells were centrifuged at 8000 rpm for 10 min (at 4°C) and the cell pellets and lysate were separated. *Lg*BSH from the cell lysate was purified using a Ni^2+^-NTA agarose column, and the purity was analyzed using 12% (wt/vol) sodium dodecylsulfate (SDS)–polyacrylamide gel electrophoresis (PAGE). The molecular weight of purified *Lg*BSH was further confirmed by a Western blot analysis, using a primary antibody against His-Tag (1:5000) and a horseradish peroxidase (HRP)-conjugated anti-mouse secondary antibody (1:10000). The protein concentration was estimated using the Bradford method (Protein Assay Kit, Bio-Rad).

### BSH Activity

Bile salt hydrolase activity was measured by the method described in Wang et al. (2012) with some modifications. Briefly, a 200-μl reaction mixture containing 178 μl of sodium-phosphate buffer (0.1 M, pH 6.0), 10 μl of purified recombinant *Lg*BSH, 2 μl of 1 M DTT, and 10 μl of 100 mM conjugated bile acid were mixed and incubated 37°C for 30 min. The reaction was terminated by mixing of 50 μl of the reaction mixture with an equal volume of 15% (w/v) trichloroacetic acid, and subsequently the precipitates were removed by centrifugation at 13,000 ×*g* for 10 min. To estimate concentrations of the liberated amino acids (glycine or taurine) from the conjugated bile acids, 50 μl of the above supernatant was mixed with 950 μl of Ninhydrin reagent and 100 μl of sodium-citrate buffer (0.5 M, pH 5.5). The mixture was kept in a water bath for 15 min and cooled in ice. Absorbance was measured at 570 nm and the amino acid concentration was estimated using a standard curve of either glycine or taurine based on the conjugated bile salts used. The enzyme activity was expressed as micromoles of amino acid released per minute per milligram of BSH. All experiments were performed in triplicate.

### Biochemical Characterization of *Lg*BSH

#### Substrate Specificity of *Lg*BSH

The substrate specificity of purified recombinant *Lg*BSH was determined using different glyco- or tauro-conjugated bile salts [glycocholic acid (GCA), glycodeoxycholic acid (GDCA), taurocholic acid (TCA), and taurodeoxycholic acid (TDCA)] as well as non-substrate compounds (penicillin V and ampicillin) as a substrate. The assay was performed as described above.

#### Effect of pH and Temperature on *Lg*BSH Activity

To identity the optimum temperature for *Lg*BSH activity, a standard reaction mixture (200 μl) was incubated at different temperature ranges (20–90°C). Similarly, a suitable pH was optimized by performing the enzyme assay at various pH values (pH 3–9). The pH was adjusted by replacing the buffer in the reaction mixture, i.e., sodium acetate buffer (pH 3–6), phosphate buffer (pH 7), and glycine-NaOH buffer (pH 8 and 9). Each experiment was performed in triplicate.

#### Effects of Reducing Agents, Surfactants, Solvents, and Enzymes on *Lg*BSH Activity

The effects of various reducing agents (DTT, β-ME, and EDTA; 10 mM each), surfactants (Triton X-100 and SDS; 10 mM each and 0.5% Tween 80), and solvents (chloroform, ethanol, acetone, methanol, isopropanol, and butanol; 10% v/v each) on *Lg*BSH activity were demonstrated. Briefly, 200 μl of the reaction mixture was separately added to various reducing agents, surfactants, and solvents and incubated for 30 min at 25°C. After incubation, an enzyme assay was performed using GCA as a substrate, and each experiment was performed in triplicate.

The effects of different enzymes (i.e., lysozyme, proteinase K, pepsin, α**-**amylase, lipase, and catalase) on *Lg*BSH activity were studied. The reaction mixture (200 μl) was treated with 2 mg/ml of each enzyme and incubated for 30 min at 25°C. The enzyme activity was evaluated after incubation as described earlier and untreated *Lg*BSH served as a control. Each experiment was performed in triplicate.

#### Inhibitory Effects of Feed Additives on *Lg*BSH Activity

To identify suitable candidates for a BSH inhibitor, various commonly used animal feed additives were screened, which including metal ions, AGPs, and other recently identified BSH inhibitors. *Lg*BSH was pre-incubated with various metal ions (i.e., CuCl_2_, CuSO_4_, MnCl_2_, MnSO_4_, MgCl_2_, MgSO_4_, ZnCl_2_, ZnSO_4_, CaCl_2_, NaHIO_3_, and KIO_3_) at a concentration of 5 mM for 30 min at 37°C (Wang et al., 2012). Similarly, AGPs, including penicillin V, ampicillin, oxytetracycline, doxycycline hydrochloride, neomycin, erythromycin, and lincomycin, were used at a concentration of 5 mM (Wang et al., 2012; Smith et al., 2014). Apart from these feed additives, several other compounds were also recently screened as BSH inhibitors which included riboflavin (Smith et al., 2014) and ascorbic acid; hence we also tested their inhibitory efficiencies against *Lg*BSH at the concentration of 5 mM. The inhibitory efficiency of inhibitors on *Lg*BSH activity was tested and calculated by dividing the mean activity of the control (without inhibitor) by the treatment group (with inhibitor).

#### Homology Modeling of *Lg*BSH

To determine the 3D structure of *Lg*BSH, the protein sequence was blasted against the protein data bank (PDB) database. The online software, Protein Homology/analogY Recognition Engine V 2.0^4^ (Phyre2), was used to predict the homologous structure of *Lg*BSH using the intensive mode (Kelley et al., 2015). Bumps were removed from the modeled protein and missing side chain atoms were added using the ‘WHAT IF Web Interface.’^5^ The predicted protein structure was validated using protein structure validation (PSVS) tool^6^. Ramachandran plot was used to analyze the structure quality. The modeled *Lg*BSH protein structures were visualized, and images were rendered using UCSF-Chimera software (Pettersen et al., 2004). Residues involved in substrate binding were identified using the computed atlas of the surface topography of proteins (CASTP) (Dundas et al., 2006) and the SiteHound-web server^7^ (Hernandez et al., 2009). Then, predicted residues were compared with the templates. These residues were used to predict putative binding sites for ligands during the docking analysis.

#### Docking Analysis

To perform the molecular docking studies, SwissDock, a small protein molecule docking web service based on EADock DSS (Fast docking using the CHARMM force field with EADock DSS) was used (Grosdidier et al., 2011). Ligands including GCA (C_26_H_43_NO_6_), GDCA (C_26_H_43_NO_5_), TCA (C_26_H_45_NO_7_S), penicillin V (PenV: C_16_H_18_N_2_O_5_S), and riboflavin (C_17_H_20_N_4_O_6_) were obtained from the ZINC database^8^ and were used in the docking analysis. Ligand structures are shown in Supplementary Figure S3. The root mean square deviation (RMSD) was used to identify the best docked complexes. The predicted docking clusters were analyzed using UCSF-Chimera.

## Results

### Identification of the BSH Gene from *L. gasseri* FR4

In our previous study, BSH activity of *L. gasseri* FR4 was demonstrated using a direct plate assay method which exhibited halos of precipitated deoxycholate on MRS-TDC agar due to the hydrolysis of TDC (Parveen Rani et al., 2016). Based on the BLAST results, a putative BSH gene (981 bp) encoding a 326-amino acid (aa) choloylglycine hydrolase family (EC 3.5.1.24) protein was identified from the genome of *L. gasseri* (NC_008530.1). The theoretical molecular weight and isoelectric point of the putative BSH were, respectively, estimated to be 36.69 kDa and 5.18 using the ExPASy analysis tool^9^. The deduced amino acid sequence of the putative *Lg*BSH was aligned with amino acid sequences of available BSH crystal structure sequences from different bacterial species. *Lg*BSH shared a sequence identity of 54% with *Enterococcus faecalis* BSH (*Ef*BSH; 4WL3), 47% with *L. salivarius* BSH (*Ls*BSH; 5HKE), 38% with *Clostridium perfringens* BSH (*Cp*BSH; 2RLC), and 37% with *Bifidobacterium longum* BSH (*Bl*BSH; 2HEZ). *Lg*BSH also shared the lowest identity of 30% with penicillin V acylase (PVA) of *Lysinibacillus sphaericus* (2PVA), since though both enzymes belong to the choloylglycine hydrolase family.

**FIGURE 1:**
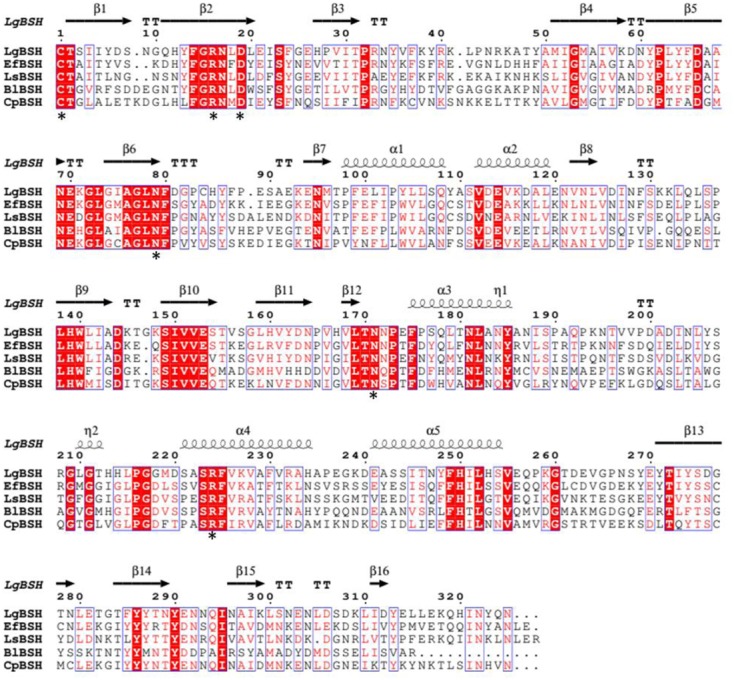
Multiple sequence alignment of bile salt hydrolase (BSH) amino acid sequences from different bacterial species. The protein sequences were aligned with Clustal Omega (http://www.ebi.ac.uk/Tools/msa/clustalo/) software and identical residues were identified by ESPript 3.0 (http://espript.ibcp.fr/ESPript/ESPript/). The identical residues are shown in red background and the similar residues are shown as red color. The conserved residues (Cys-1, Arg-16, Asp-19, Asn-79, Asn-171, and Arg-224) were marked as black asterisk (^∗^).

The multiple sequence alignment results revealed similarities of conserved amino acid residues among all of the selected BSHs, which included a catalytic nucleophile residue, Cys1, and other conserved amino acids, such as Arg16, Asp19, Asn79, Asn171, and Arg224 (amino acids were numbered from cysteine) (**Figure 1**). Rossocha et al. (2005), Kumar et al. (2006), and Xu et al. (2016) reported that Cys1 plays an important role in the activity of BSH. This further confirmed that the identified gene belonged to BSH.

The evolutionary relationship of *Lg*BSH with other bacterial choloylglycine hydrolase family proteins (BSH/PVA) was inferred using a UPGMA phylogenetic analysis. Evolutionary distances were computed using the Poisson correction method and are in units of the number of amino acid substitutions per site. The analysis involved 37 aa sequences of BSH and PVA from various bacterial taxa. All positions containing gaps and missing data were eliminated. There were 266 positions in total in the final dataset. The phylogenetic tree showed that BSHs of *Lactobacillus* spp. were distinct from the PVA, which has an evolutionary relationship with BSH. *Lg*BSH was closely related to BSHs of *E. faecalis, C. perfringens*, and *L. salivarius* (Supplementary Figure S4).

### Expression and Purification of Recombinant *Lg*BSH

**FIGURE 2:**
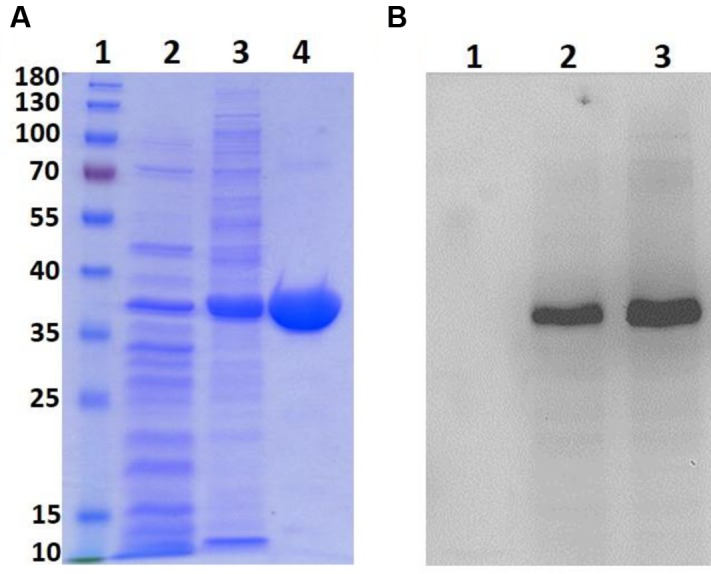
Expression and purification of *Lg*BSH. SDS–PAGE analysis of *Lg*BSH **(A)**. Lane1: Marker (PageRuler^TM^ Prestained Protein Ladder, 10–180 kDa), Lane2: cell lysate of *Escherichia coli* BL21 wild type, Lane3: cell lysate of *E. coli* BL21 expressing *Lg*BSH, Lane4: purified *Lg*BSH. Western blotting analysis of *Lg*BSH using anti-His antibody **(B)**. Lane1: cell lysate of *E. coli* BL21 wild type, Lane2: cell lysate of *E. coli* BL21 expressing *Lg*BSH, Lane3: purified *Lg*BSH.

The expression vector pET21b (+) harboring the *Lg*BSH-encoding gene was transformed into *E. coli* BL21 (DE3) to overexpress *Lg*BSH. A recombinant *Lg*BSH protein band was observed after IPTG induction on SDS–PAGE at around 37 kDa, which is the calculated molecular mass of *Lg*BSH (**Figure 2A**, lane 3). A single band was observed at 37 kDa after purification with Ni^2+^-NTA agarose column, thus confirming the homogeneity and purity of *Lg*BSH (**Figure 2A**, lane 4). The Western blot analysis using anti-His antibodies also confirmed the molecular weight of purified *Lg*BSH (**Figure 2B**).

### Biochemical Characterization of *Lg*BSH

#### Substrate Specificity of *Lg*BSH

**FIGURE 3:**
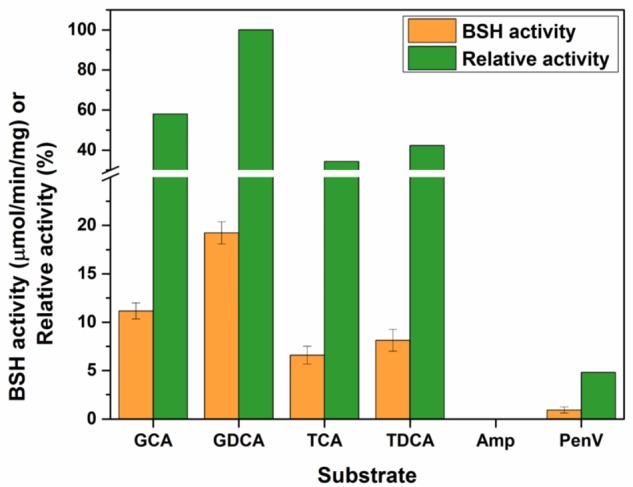
Determining the substrate specificity of *Lg*BSH using various bile salts as well as non-substrate compounds. The BSH activity was expressed as the μmol/min/mg. All the experiments were performed in triplicate and mean ± SD values are represented.

To determine the substrate specificity of purified *Lg*BSH, four major glyco- or tauro-conjugated bile salts were used along with two non-substrate compounds (i.e., penicillin V and ampicillin). The highest *Lg*BSH activity was observed with GDCA as a substrate and was set as 100% activity. *Lg*BSH showed greater hydrolysis toward glyco-conjugated bile salts (GCA and GDCA) than to tauro-conjugated (TCA and TDCA) bile salts (**Figure 3**). However, very weak activity (4.78%) was observed when using penicillin V as a substrate, and no activity was detected with ampicillin (**Figure 3**). These results further confirmed that the identified *Lg*BSH was not PVA.

#### Effects of pH and Temperature on *Lg*BSH Activity

**FIGURE 4:**
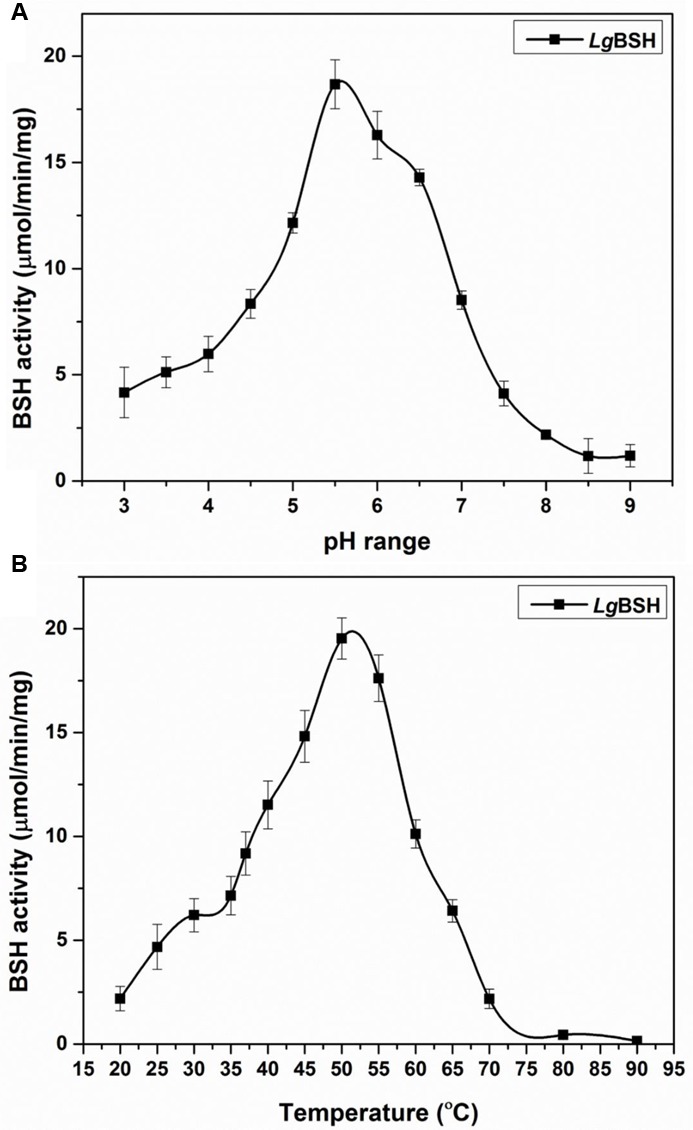
Effect of various pH **(A)** and temperature **(B)** on *Lg*BSH activity. The assay was performed using glycocholic acid (GCA) as a substrate and activity was expressed as the μmol/min/mg. All the experiments were performed in triplicate and mean ± SD values are represented.

To determine the optimal pH for maximal *Lg*BSH activity, various pH values (pH 3–9) were used and optimal *Lg*BSH activity was observed in the range of pH 5.5–6.5. The maximal BSH activity (18.68 ± 1.15 μmol/min/mg) was observed at pH 5.5. Activities decreased at lower and higher pH values (**Figure 4A**). The optimal temperature for *Lg*BSH activity was determined using various temperature values (20–90°C). The maximal BSH activity was observed at 52°C and enzyme activity sharply declined with increasing temperatures (**Figure 4B**).

#### Effects of Various Reducing Agents, Surfactants, Solvents, and Enzymes on *Lg*BSH Activity

The effects of various reducing agents on *Lg*BSH were analyzed. *Lg*BSH activity increased by about 18% when the enzyme was treated with 10 mM DTT (118.06%). However, the activity reduced by 2 and 4%, when treated with β-mercaptoethanol (98.18%) and EDTA (96.14%), respectively (**Table 1**). Surfactants such as Triton X-100 and Tween 80 significantly increased *Lg*BSH activity by 43 and 28%, respectively. Generally, surfactants or detergents possibly interact with the surfaces of enzymes, thereby enhancing the solubility and stability of proteins (Avinash et al., 2015). On the other hand, SDS dramatically reduced BSH activity by 93% at a concentration of 10 mM.

**Table 1:** Effects of various reducing agents, surfactants, solvents, and enzymes on purified *Lactobacillus gasseri* bile salt hydrolase (*Lg*BSH) activity.

Treatment	Compound	Relative activity (%)^a^
Control		100% l
Reducing agents	DTT^b^	118.06 ± 0.31 l
(10 mM)	β-Mercaptoethanol	98.18 ± 0.83 l
	EDTA^b^	96.14 ± 1.16 l
Surfactants	Triton X-100	143.12 ± 1.47 l
(10 mM or 0.5%)	Tween 80	128.19 ± 0.82 l
	SDS^b^	2.86 ± 0.19 l
Solvents	Chloroform	98.91 ± 1.16 l
(10% [vol/vol])	Ethanol	86.16 ± 1.67 l
	Acetone	108.71 ± 1.05 l
	Methanol	69.15 ± 2.15 l
	Isopropanol	141.21 ± 1.26 l
	Butanol	118.20 ± 2.19 l
Enzymes	Lysozyme	91.35 ± 0.92 l
	Proteinase K	3.49 ± 0.19 l
	Pepsin	15.16 ± 1.68 l
	α**-**Amylase	98.41 ± 1.24 l
	Lipase	101.19 ± 0.62 l
	Catalase	98.13 ± 0.53 l

*^a^The relative activity percentage was calculated based on the control with no treatment, and the assay was performed using glycocholic acid as a substrate.*

*^b^DTT, dithiothreitol; EDTA, ethylenediaminetetraacetic acid; SDS, sodium dodecylsulfate.*

Organic solvents such as isopropanol, butanol, and acetone, respectively, enhanced *Lg*BSH activities by 41, 18, and 8% when the enzyme was treated with a 10% (v/v) concentration. Several solvents such as methanol, ethanol, and chloroform significantly decreased the enzyme activity. Among them, treatment with methanol drastically decreased the enzyme activity by 31% (**Table 1**).

The influences of various enzymes on *Lg*BSH activity was investigated by treating BSH with appropriate enzymes. Proteinase K and pepsin, respectively, decreased *Lg*BSH activity by 97 and 85%, thus confirming that proteinase K might degrade the BSH enzyme. However, enzymes like lysozyme, α**-**amylase, lipase, and catalase did not affect *Lg*BSH activity. This was due to the inefficiencies of these enzymes on *Lg*BSH (**Table 1**).

#### Inhibitory Effects of Feed Additives on *Lg*BSH Activity

Various commonly used feed additives and AGPs were screened to identify a suitable *Lg*BSH inhibitor. Among the tested compounds, more than 95% inhibition was observed with riboflavin, NaHIO_3_, CuCl_2_, penicillin V, KIO_3_, doxycycline hydrochloride, ampicillin, and oxytetracycline (**Table 2**). The lowest inhibitory percentage (<50%) was observed with erythromycin, ZnSO_4_, MgCl_2_, ascorbic acid, MgSO_4_, CuCl_2_, and lincomycin. Among all of the tested inhibitors, riboflavin might be a good candidate for application in animal feed.

**Table 2:** Inhibitory effects of various compounds on *L. gasseri* bile salt hydrolase (*Lg*BSH) activity.

Compound type	Compound name	Inhibition %^a^
Metal ion or feed additive	CuCl_2_	98.13
	CuSO_4_	94.36
	MnCl_2_	76.42
	MnSO_4_	73.98
	MgCl_2_	36.14
	MgSO_4_	32.69
	ZnCl_2_	54.21
	ZnSO_4_	39.64
	CaCl_2_	18.64
	NaHIO_3_	98.26
	KIO_3_	97.64
Antibiotics used as growth promoters (AGPs)	Penicillin V	97.84
	Ampicillin	96.39
	Oxytetracycline	95.97
	Doxycycline hydrochloride	97.51
	Neomycin	82.93
	Erythromycin	41.62
	Lincomycin	18.58
Potential inhibitors	Riboflavin	98.31
	Ascorbic acid	32.96

*^a^The inhibition percentage was calculated by dividing the mean activity of the control (without an inhibitor) and treatment (with an inhibitor). The assay was performed with 5 mM of each inhibitor, and glycocholic acid was used as a substrate.*

#### Homology Modeling of *Lg*BSH

**FIGURE 5:**
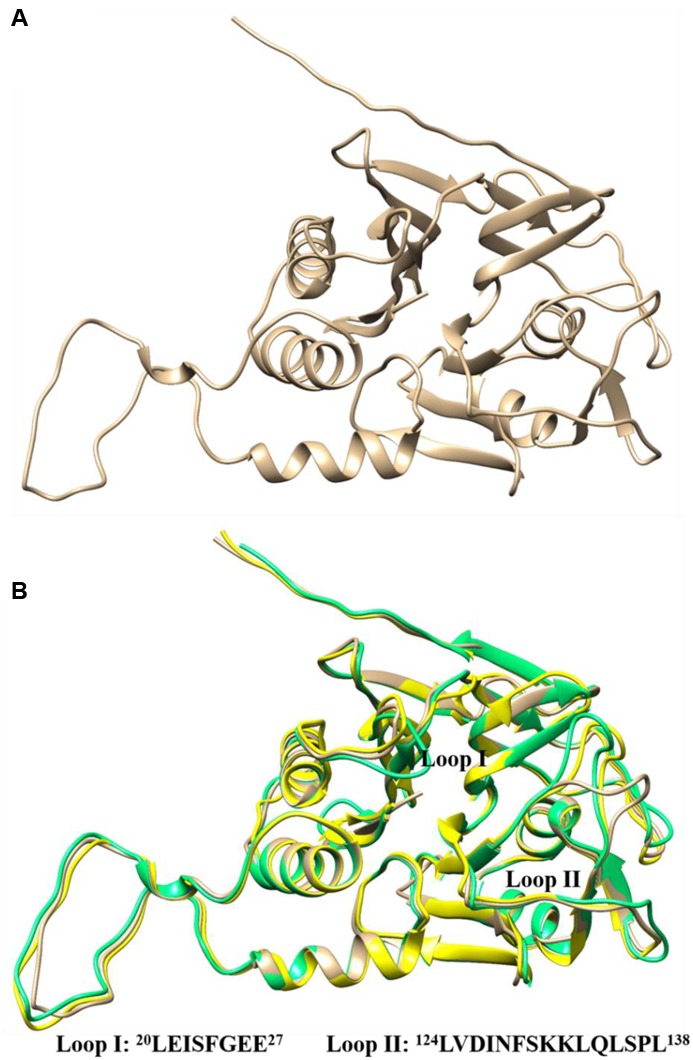
Three-dimensional structure of *Lg*BSH predicted by homology modeling using *Enterococcus faecalis* BSH (4WL3) as template **(A)**. Three-dimensional superimposed structures of *Lg*BSH (tan) with their closest homology structures *Ef*BSH (4WL3) (yellow) and *Ls*BSH (5HKE) (spring green) using UCSF-Chimera. The sequences of loops (I and II) present in the substrate-binding pockets were represented in the figure **(B)**.

Protein BLAST search of *Lg*BSH showed highest identity of about 54% with the BSH of *E. faecalis* (4WL3), and 47% identity with the BSH of *L. salivarius* (5HKE). The homology model of *Lg*BSH was predicted with the Phyre2 algorithm using *Ef*BSH (4WL3) as a template (**Figure 5A**), since it had a 100% confidence level (Supplementary Figure S5). The superimposed structure of *Ef*BSH, *Ls*BSH with the *Lg*BSH revealed similarities among their structures and their catalytic active sites contains nucleophile residue (Cys1) (**Figure 5B**). Residues involved in catalysis were identified based on the superimposed structure, including Cys1, Arg16, Asp19, Asn79, Asn171, and Arg224. However, residues in the substrate-binding pocket were conservatively replaced. Two loops were identified near the substrate-binding pockets: loop I contained 8 aa from 20 to 27 (LEISFGEH), and loop II contained 15 aa from 124 to 138 (LVDINFSKKLQLSPL). The secondary structure prediction of *Lg*BSH using PSIPRED V 3.3 revealed that *Lg*BSH contains 14.11% of α-helix, 60.12% of random coil, and 25.76% of extended strand (Supplementary Figure S6). The secondary structural pattern of *Lg*BSH was similar to *Ef*BSH.

The PSVS analysis results of predicted *Lg*BSH structure suggested that the model was of a good quality. The Ramachandran plot analysis showed that 91.6% of residues lies in the most favored regions; 8.1 and 0.4% of residues lies in allowed and disallowed regions, respectively (Supplementary Figure S7). The Procheck G-factor (all dihedral angles) was found to be -0.19, and the VERIFY-3D results showed that 85.54% of residues had an average 3D-1D score of >0.2, thus confirming that the predicted *Lg*BSH structure contained no conformational errors (Supplementary Figure S8). The overall *Z*-score of the ProSA analysis was -6.57 (Supplementary Figure S9), which is in the range of scores typically found for native proteins of similar sizes (Wiederstein and Sippl, 2007). The overall quality factor of the predicted *Lg*BSH model was calculated by ERRAT, and results showed 90.033, which further confirmed the quality of the predicted structure (Supplementary Figure S10). The RMSDs of bond angles and bond lengths were 2.2° and 0.019 Å, respectively. The modeled *Lg*BSH protein structure was submitted to Protein Model Data Base^10^ (PMDB) with the model id PM0080976.

The CASTp analysis revealed residues involved in substrate binding. On the whole, 19 possible residues were identified and compared to binding pocket residues of the template protein (Supplementary Figure S11). The approximate binding site volume of *Lg*BSH was found to be 551.7 Å^3^. The SiteHound analysis revealed that the total interaction energy (TIE) of the substrate-binding pocket was -480.02 kcal/mol with the lowest and highest interaction energies of -22.34 and -8.96 kcal/mol, respectively.

#### Molecular Docking Studies to Identify the Substrate Specificity and Inhibitors

**FIGURE 6:**
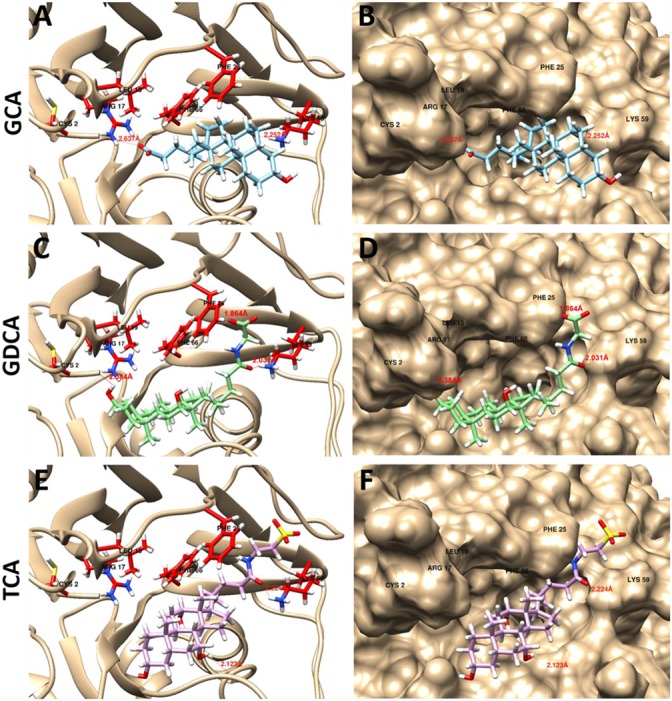
Docking analysis of GCA **(A,B)**, glycodeoxycholic acid (GDCA) **(C,D)**, and taurocholic acid (TCA) **(E,F)** with *Lg*BSH to determine the substrate specificity and identify the hydrogen bonding between enzyme and substrate. Docking was performed using SwissDock and the outputs were analyzed using UCSF-Chimera.

*Lg*BSH had a substrate preference toward glyco-conjugated bile salts (GCA and GDCA), compared to tauro-conjugated bile salts. Hence, to demonstrate the substrate specificity, GCA, GDCA, and TCA were docked with *Lg*BSH, and their hydrogen bonding interactions with the enzyme were identified (**Figure 6**). Cys2 and Lys59 (amino acids were numbered from methionine) residues in the substrate-binding pocket of *Lg*BSH formed hydrogen bonds with GCA, with respective bond lengths of 2.637 and 2.252 Å (**Figures 6A,B**). Similarly, Cys2, Phe25, and Lys59 interacted with GDCA via hydrogen bonding with respective bond lengths of 2.384, 1.864, and 2.031 Å (**Figures 6C,D**). On the other hand, TCA formed hydrogen bonds with Lys59 and Gln135 with respective bond lengths of 2.224 and 2.123 Å, (**Figures 6E,F**). However, the binding energy of GCA was found to be lower (-8.46 kcal/mol) than that of GDCA (-6.81 kcal/mol) and TCA (-7.91 kcal/mol), thus further confirming the binding strength of GCA over other substrates.

**FIGURE 7:**
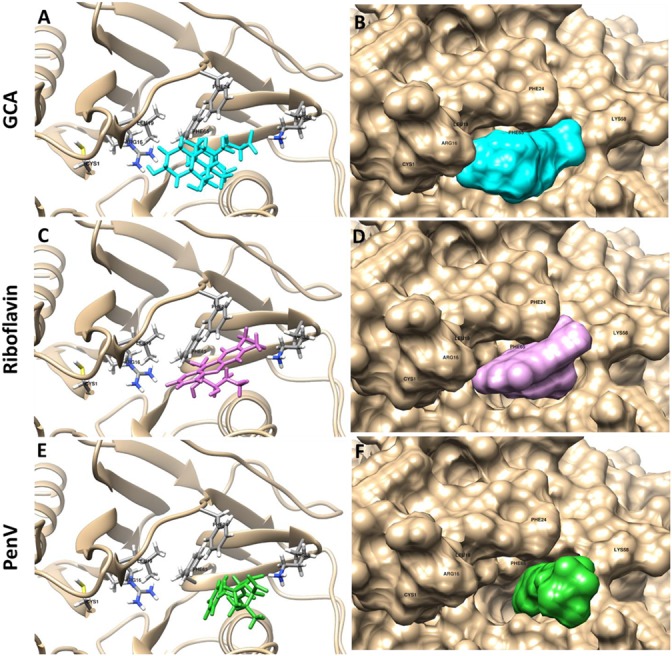
Docking analysis of GCA (**A,B**: cyan), riboflavin (**C,D**: pink), and penicillin V (**E,F**: green) with *Lg*BSH to determine the inhibitory effects. Docking was performed using SwissDock and the outputs were analyzed using UCSF-Chimera.

Based on previous experimental data, we found that riboflavin and penicillin V, respectively, inhibited *Lg*BSH activity by 98.31 and 97.84%. To further investigate the mechanism behind this inhibition and support our experimental data, both substrate (GCA) and inhibitors (riboflavin and penicillin V) were docked with the modeled protein structure using the SwissDock algorithm. All ligands showed favorable binding energies, among which the substrate GCA had the lowest binding energy of -8.46 kcal/mol, while on the other hand, inhibitors, i.e., penicillin V and riboflavin, had relatively higher binding energies of -7.38 and -6.25 kcal/mol, respectively (**Figure 7**).

## Discussion

Bile salt hydrolase enzymes of several LAB species play a central role in the lipid metabolism of hosts. During the past several decades, probiotic bacteria with BSH activity were used to alleviate cholesterol levels in humans and animals (Jones et al., 2004). Several authors have previously identified and crystallized BSH enzymes from various bacterial species including *C. perfringens* (Rossocha et al., 2005), *B. longum* (Kumar et al., 2006), *L. salivarius* (Xu et al., 2016), and *E. faecalis*. Kumar et al. (2013) identified and cloned a gene encoding the BSH enzyme from *L. fermentum* NCDO394. The nucleotide sequence of the putative BSH gene contained an open reading frame (ORF) of 978 nucleotides encoding a predicted protein of 325 aa. They reported that the deduced BSH protein had significant similarity to the penicillin V amidases of other *Lactobacillus* spp. Wang et al. (2012) identified a BSH gene from the genome of *L. salivarius* NRRL B-30514, a chicken isolate. Similarly, we have identified a putative BSH gene (981 bp) encoding 326 aa from the genome of *L. gasseri* FR4. The molecular mass of purified *Lg*BSH was found to be 37 kDa on SDS–PAGE and was further confirmed by a Western blot analysis (**Figures 2A,B**). This result is further supported by several publications, where the molecular mass of the BSH enzyme was reported as 37 kDa on SDS–PAGE (Wang et al., 2012; Kumar et al., 2013; Jayashree et al., 2014).

Generally, BSH enzymes have a broad range of substrate specificity toward glyco- and tauro-conjugated bile salts (Lambert et al., 2008; Oh et al., 2008). Purified *Lg*BSH exhibited substrate specificity toward glycine-conjugated bile salts (GCA and GDCA) and in particular, maximal activity was observed on GDCA (**Figure 3**). However, the activity was inhibited when using non-substrate compounds (i.e., ampicillin and penicillin V) as a substrate. Kumar et al. (2013) studied the characteristics of recombinant (r) BSH of *L. fermentum* NCDO394 and identified that purified rBSH enzyme hydrolyzed six major bile substrates, with a special preference toward glycine-conjugated bile salts and exhibited maximal activity against GCA. Previous studies also confirmed that BSHs from *Lactobacillus* spp., including *L. salivarius* (Wang et al., 2012), *L. plantarum* CGMCC 8198 (Gu et al., 2014), and BSH-C of *L. johnsonii* PF01 (Chae et al., 2013), had a substrate specificity toward glyco-conjugated bile salts. Biochemical characterization studies revealed that the pH and temperature optima of *Lg*BSH were, respectively, found to be 5.5 and 52°C. Our results are in accordance with the results of Wang et al. (2012), in which the BSH from *L. salivarius* showed maximal activity at 41°C and pH 5.4. Kumar et al. (2013) also found that the optimal pH and temperature for enzymatic activity of BSH from *L. fermentum* NCDO394 were pH 6.0 and 37°C, respectively.

The evolution of protein structures is relatively slower than the evolution of nucleic acids, thus providing stability to protein structures. Proteins with similar sequences have retained identical structure during evolution, and proteins from distantly related organisms with less sequence similarity also have similar protein structures and retain similar functions (Kaczanowski and Zielenkiewicz, 2010; Chae et al., 2013). Although BSHs of various species had the highest sequence divergence, the protein structures were still very similar. Hence, homology modeling is an ideal approach to obtain the putative structure of the identified BSH enzyme, thus helps to understand the characteristics of the substrate-binding pocket and other functions of the enzyme. Superimposing the structure of *Lg*BSH onto that of *Ef*BSH revealed similarities of protein structures and binding pockets. Since BSH and PVA evolved from the same origin, both enzymes have the same catalytically important active site residues (Cys1, Arg16, Asp19, Asn79, Asn171, and Arg224) except for Asn79, where PVA uses tyrosine instead of asparagine (Rossocha et al., 2005). Recently, Xu et al. (2016) resolved the crystal structure of the BSH enzyme from *L. salivarius*, which had 47% sequence similarity with *Lg*BSH. Hence, we used this crystal structure to characterize our *Lg*BSH. Two loops (loops I and II) were identified from the substrate-binding pocket of *Lg*BSH. According to Xu et al. (2016), residues Leu133 (Leu133 in *Ls*BSH and Leu132 in *Ef*BSH) and Phe129 (Phe129 in *Ls*BSH and Phe128 in *Ef*BSH) in loop II might contribute to limiting the spatial configuration via condensing the entrance of the substrate-binding pocket. Similarly, the Phe24 (Tyr23 in *Ls*BSH and Tyr23 in *Ef*BSH) residue in loop I along with the Phe64 (Phe64 in *Ls*BSH and Phe64 in *Ef*BSH) residue might allow the substrate to intensely dock into the substrate-binding pocket, and thus may also be involved in enzyme–substrate interactions (Rossocha et al., 2005; Xu et al., 2016). The mode of substrate binding is dependent on the loops surrounding the active site, which also define the volume of the site (**Figure 5B**). Polar complementarity toward conjugated bile acids was also proposed as an important facet of substrate specificity (Chand et al., 2017). *Lg*BSH exhibited substrate specificity toward glyco-conjugated bile salts and had the lowest binding energy. Similarly, BSHs from most bacterial species had substrate preferences toward glyco-conjugated bile salts rather than tauro-conjugated ones. The major reasons for these phenomena include, steric hindrance caused by a sulfur atom of taurine and an abundance of glyco-conjugated bile salts in nature (Chand et al., 2017).

Recent epidemiological studies suggests that the use of AGPs is related to the emergence of antibiotic-resistant bacteria which may be transmitted to humans and cause significant threats to food safety and public health (Lin, 2014). However, a ban on AGP usage would create challenges for the animal feed and feed-additive industries. Several products, viz., probiotics, prebiotics, and organic acids, are used to change the intestinal microbiota to improve animal health and production. Studies using swine and poultry fostered understanding of the relationships between AGP supplementation and GIT bacterial compositions. Results of several studies proved that AGPs have created bacterial shifts, have altered the microbial diversity of the intestines, and suggested that certain GIT populations might be more related to animal growth (Danzeisen et al., 2011; Kim et al., 2012; Lin, 2014). However, probiotic feed supplements lead to decreased body mass gains in treated animals. This can be overcome by feeding animals with suitable BSH inhibitors along with the probiotic feed. A high purity of BSH enzymes is necessary to screen BSH inhibitors and study the substrate specificity of the enzyme. Hence, we have purified the BSH enzyme using His tag and used screened various compounds to identify potential BSH inhibitors. The results suggested that CuCl_2_ decreased BSH activity by 98.13% among all of the tested metal ions (**Table 2**). Wang et al. (2012) demonstrated that copper (CuCl_2_) and zinc (ZnSO_4_) inhibited BSH activities by 98.1 and 89.5%, respectively. Conversely, we found that inhibition of *Lg*BSH activity by zinc (ZnCl_2_ or ZnSO_4_) was not significant. We also tested the inhibitory effects of currently used AGPs and found that all tested antibiotics (except erythromycin and lincomycin) had significant inhibitory effects on *Lg*BSH. However, the long-term use of these metal ions and AGPs might affect animal health, increase production costs and accumulation of metal ions in treated animals. Recently, Smith et al. (2014) performed high-throughput screening (HTS) to identify safe and cost-effective BSH inhibitors and reported that riboflavin and phenethyl caffeate can act as potential BSH inhibitors. These results were further supported by Lin et al. (2014), who reported that riboflavin and CAPE could inhibit BSH activity. Hence, we used riboflavin as a novel inhibitor and found that *Lg*BSH activity was reduced by up to 98.31% (**Table 2**). Riboflavin (or vitamin B2) play key roles in various intracellular (i.e., energy metabolism) and extracellular (i.e., quorum sensing and extracellular electron transfer) processes in bacteria. Hence, riboflavin has been used as a feed additive for several animals to overcome vitamin B2 deficiencies (Smith et al., 2014; Gutiérrez-Preciado et al., 2015). The Food and Drug Administration (FDA) of United States of America (USA) has already approved riboflavin as a feed additive, since it has well-known metabolic functions (Smith et al., 2014). The addition of riboflavin along with the probiotic *L. gasseri* FR4 can serve as a BSH inhibitor and also increase survival rates of probiotic bacteria. Gutiérrez-Preciado et al. (2015) performed extensive analyses on riboflavin transporters across all bacterial strains and found that *L. gasseri* has its unique transporter for riboflavin. Stahly et al. (2007) conducted a swine study and reported that dietary supplementation with riboflavin (20 mg/kg feed) significantly enhanced the body weight and feed efficiency in pigs. The current studies suggested this might be due to inhibition of the BSH enzyme produced by probiotic microorganisms present in the swine’s intestines. To further unravel the mechanism behind the inhibitory effect of riboflavin, we performed a molecular docking analysis using the predicted *Lg*BSH structure. Results showed that both substrate and inhibitors (riboflavin and penicillin V) could bind to the substrate-binding pocket of *Lg*BSH with almost the same binding affinity/energy (**Figure 7**). This might have been due to the inverse mode of binding of riboflavin and penicillin V. This study provides some new insights into the inhibitory effects of several potential BSH inhibitors, and these might serve as alternatives for AGPs.

## Conclusion

In this study, we identified and characterized a novel BSH enzyme from probiotic *L. gasseri* FR4. *Lg*BSH showed greater hydrolysis toward glyco-conjugated bile salts than to tauro-conjugated bile salts, and no hydrolysis was observed on penicillin V. The homology modeling studies revealed the 3D structure of *Lg*BSH, which has a structural similarity with previously identified BSH enzymes. *Lg*BSH activity was dramatically inhibited by riboflavin and confirmed by molecular docking analysis. Riboflavin had almost the same binding energy as GCA, thus helping in the inhibition of *Lg*BSH. Hence, supplementation of *L. gasseri* FR4 along with riboflavin might be used as an alternative for AGPs for poultry animals. However, detailed animal studies are necessary to further confirm the efficiency of BSH inhibitors on animal growth and performance.

## Author Contributions

AR, RR, and MA contributed with the conception and experimental design. RR, carried out all experiments. MA and RR performed the homology modeling and docking analysis. MA and RR analyzed and interpreted the results. RR written the manuscript and corrected by AR and MA. All authors performed a critical revision of the manuscript and approved the final version.

## Conflict of Interest Statement

The authors declare that the research was conducted in the absence of any commercial or financial relationships that could be construed as a potential conflict of interest.

## Footnotes

1 http://www.ncbi.nlm.nih.gov/

2 http://www.ebi.ac.uk/Tools/msa/clustalo/

3 http://espript.ibcp.fr/ESPript/ESPript/

4 http://www.sbg.bio.ic.ac.uk/phyre2

5 http://swift.cmbi.ru.nl/servers/html/index.html

6 http://psvs-1_5-dev.nesg.org

7 http://sitehound.sanchezlab.org

8 http://zinc.docking.org/

9 http://web.expasy.org/compute_pi

10 https://bioinformatics.cineca.it/PMDB/
