# Supplementary material for: Characterization of Bile Salt Hydrolase from Lactobacillus gasseri FR4 and Demonstration of Its Substrate Specificity and Inhibitory Mechanism Using Molecular Docking Analysis
Source: Front Microbiol. 2017 May 31;8:1004. doi: 10.3389/fmicb.2017.01004 (PMC5449720; doi:10.3389/fmicb.2017.01004)
Supplement: Supplementary file 1 [file Data_Sheet_1.PDF]

Supplementary materials

Characterization of bile salt hydrolase from *Lactobacillus gasseri* FR4 and demonstration of its substrate specificity and inhibitory mechanism using molecular docking analysis

Running Title: Bile salt hydrolase from *Lactobacillus gasseri* FR4

Rizwana Parveen Rani[a], Marimuthu Anandharaj[b] and Abraham David Ravindran[a]*

[a]Department of Biology, The Gandhigram Rural Institute-Deemed University, Gandhigram-624302, Tamilnadu, India.

[b]Biodiversity Research Center, Academia Sinica, Taipei-115, Taiwan.

E-Mail: rizm_green@yahoo.com, anandharaj49@gmail.com and david_gribiology@rediffmail.com

Supplementary Figures

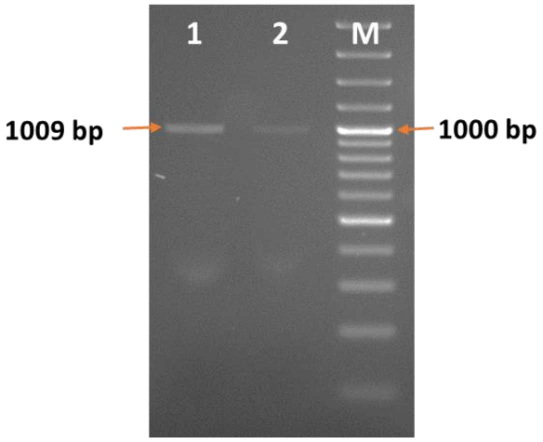

Fig. S1: Amplification of BSH gene from genomic DNA of *L. gasseri* FR4. Lane 1 and 2: BSH. M: Marker GeneRuler 100bp DNA ladder plus (Fermentas, USA).

```
pET21b(+)-LgBSH      gctagttattgctcagcggtggcagcagccaactcagcttcctttcgggctttgttagca
T7-Terminator-R      ----------------cgattgaagcagccaactcagcttcctttcgggctttgttagca
                                     ** * * *************************************

pET21b(+)-LgBSH      gccggatctcagtggtggtggtggtggtgctcgagattttgatagttaatatgttgcttt
T7-Terminator-R      gccggatctcagtggtggtggtggtggtgctcgagattttgatagttaatatgttgcttt
                     ************************************************************

pET21b(+)-LgBSH      tcaagcaattcataatcaattaacttgtcactatctaaattttcattgcttaatttaata
T7-Terminator-R      tcaagcaattcataatcaattaacttgtcactatctaaattttcattgcttaatttaata
                     ************************************************************

pET21b(+)-LgBSH      gcattaatttgattattttcatagttggtataatagaaagttccagtctccaaattagtt
T7-Terminator-R      gcattaatttgattattttcatagttggtataatagaaagttccagtctccaaattagtt
                     ************************************************************

pET21b(+)-LgBSH      ccatcagagtaaatagtatattcatatgaatttggacctacttcatctgttccttttggt
T7-Terminator-R      ccatcagagtaaatagtatattcatatgaatttggacctacttcatctgttccttttggt
                     ************************************************************

pET21b(+)-LgBSH      tgttcgacagaatgaagaatatggaaataatttgttatactacttgcctcatccttgcct
T7-Terminator-R      tgttcgacagaatgaagaatatggaaataatttgttatactacttgcctcatccttgcct
                     ************************************************************

pET21b(+)-LgBSH      tcaggtgcatgagctcgaacaaaggcaactttaacaaatcgactggctgaatccattcct
T7-Terminator-R      tcaggtgcatgagctcgaacaaaggcaactttaacaaatcgactggctgaatccattcct
                     ************************************************************

pET21b(+)-LgBSH      ccaggtaagtgatgtgtacctaaacctctactataaagattaatatcagcatcaggaacc
T7-Terminator-R      ccaggtaagtgatgtgtacctaaacctctactataaagattaatatcagcatcaggaacc
                     ************************************************************

pET21b(+)-LgBSH      acagtattttaggttgtgcaggagaaatattagcgtaatttgctaaattggttaattga
T7-Terminator-R      acagtattttaggttgtgcaggagaaatattagcgtaatttgctaaattggttaattga
                     ************************************************************

pET21b(+)-LgBSH      cttggaaattccgggttattagtcaaaacatgaactggattgtcatagacatgtaagcca
T7-Terminator-R      cttggaaattccgggttattagtcaaaacatgaactggattgtcatagacatgtaagcca
                     ************************************************************

pET21b(+)-LgBSH      cttacagttgattctacaacaattgacttaccagtcttatcagcaattaaccaatgtagc
T7-Terminator-R      cttacagttgattctacaacaattgacttaccagtcttatcagcaattaaccaatgtagc
                     ************************************************************

pET21b(+)-LgBSH      ggtgataattgtaatttttagaaaaattaatatcaaccaaattaacattttctaatgca
T7-Terminator-R      ggtgataattgtaatttttagaaaaattaatatcaaccaaattaacattttctaatgca
                     ************************************************************

pET21b(+)-LgBSH      tcttttacctcatcaactgaagcatattgactcaataaatatgggattaattcaaatgga
T7-Terminator-R      tcttttacctcatcaactgaagcatattgactcaataaatatgggattaattcaaatgga
                     ************************************************************

pET21b(+)-LgBSH      gtcatattttctttttctgctgattcggggaaatagtgacatggaccatcaaaattaagt
T7-Terminator-R      gtcatattttctttttctgctgattcggggaaatagtgacatggaccatcaaaattaagt
                     ************************************************************

pET21b(+)-LgBSH      ccagcaattcctaaccctttctcattagctgcatcaaaatataaaggatagttatcttta
T7-Terminator-R      ccagcaattcctaaccctttctcattagctgcatcaaaatataaaggatagttatcttta
                     ************************************************************

pET21b(+)-LgBSH      acgattgccatcccaatcatagcataggttgcctttctattaggtaacttccgatatttg
T7-Terminator-R      acgattgccatcccaatcatagcataggttgcctttctattaggtaacttccgatatttg
                     ************************************************************

pET21b(+)-LgBSH      aacacataatttctcggtgtaattactgggtgttcaccaaaagaaatttctaaatctaaa
T7-Terminator-R      aacacataatttctcggtgtaattactgggtgttcaccaaaagaaatttctaaatctaaa
                     ************************************************************

pET21b(+)-LgBSH      ttacgcccaaaataatgttgaccgtttgaatcataaataattgaggtacaggatccccgac
T7-Terminator-R      ttacgcccaaaataatgttgaccgtttgaatcataaataattgaggtacaggatccccgac
                     ************************************************************

pET21b(+)-LgBSH      ccatttgctgtccaccagtcatgctagccatatgtatatctccttcttaaagttaaacaa
T7-Terminator-R      ccatttactgtactc---------------------------------------------
                     ****** **** * *

pET21b(+)-LgBSH      aattatttctaga
T7-Terminator-R      -------------
```

Fig. S2: Sequencing results of *Lg*BSH encoding gene using T7-R primer. The amplified *Lg*BSH gene does not contains any mutations. The restriction enzymes *Xba*I and *Bam*HI were highlighted in green and red colour, respectively.

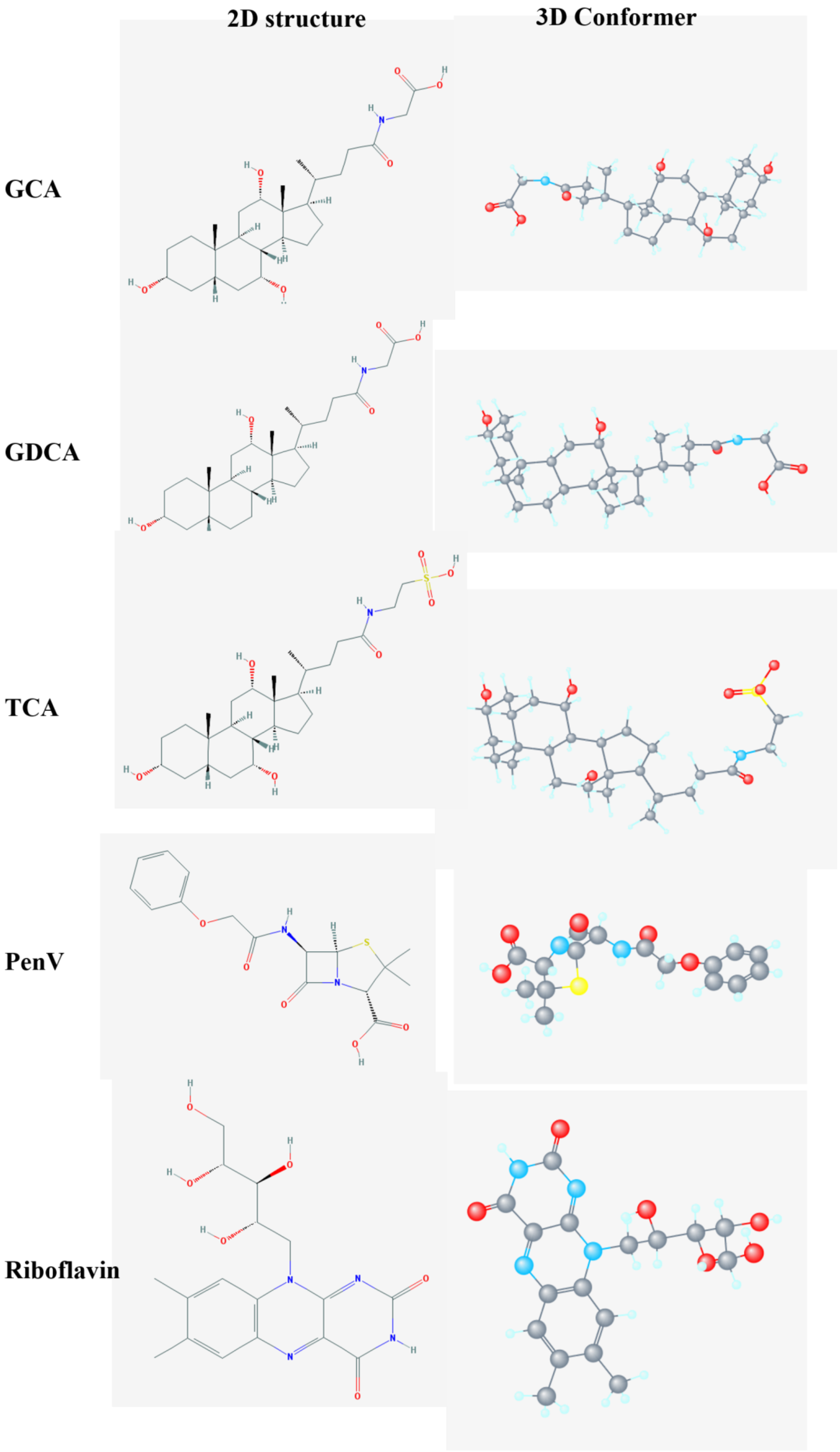

Fig. S3: 2D and 3D structures of ligands used in this study

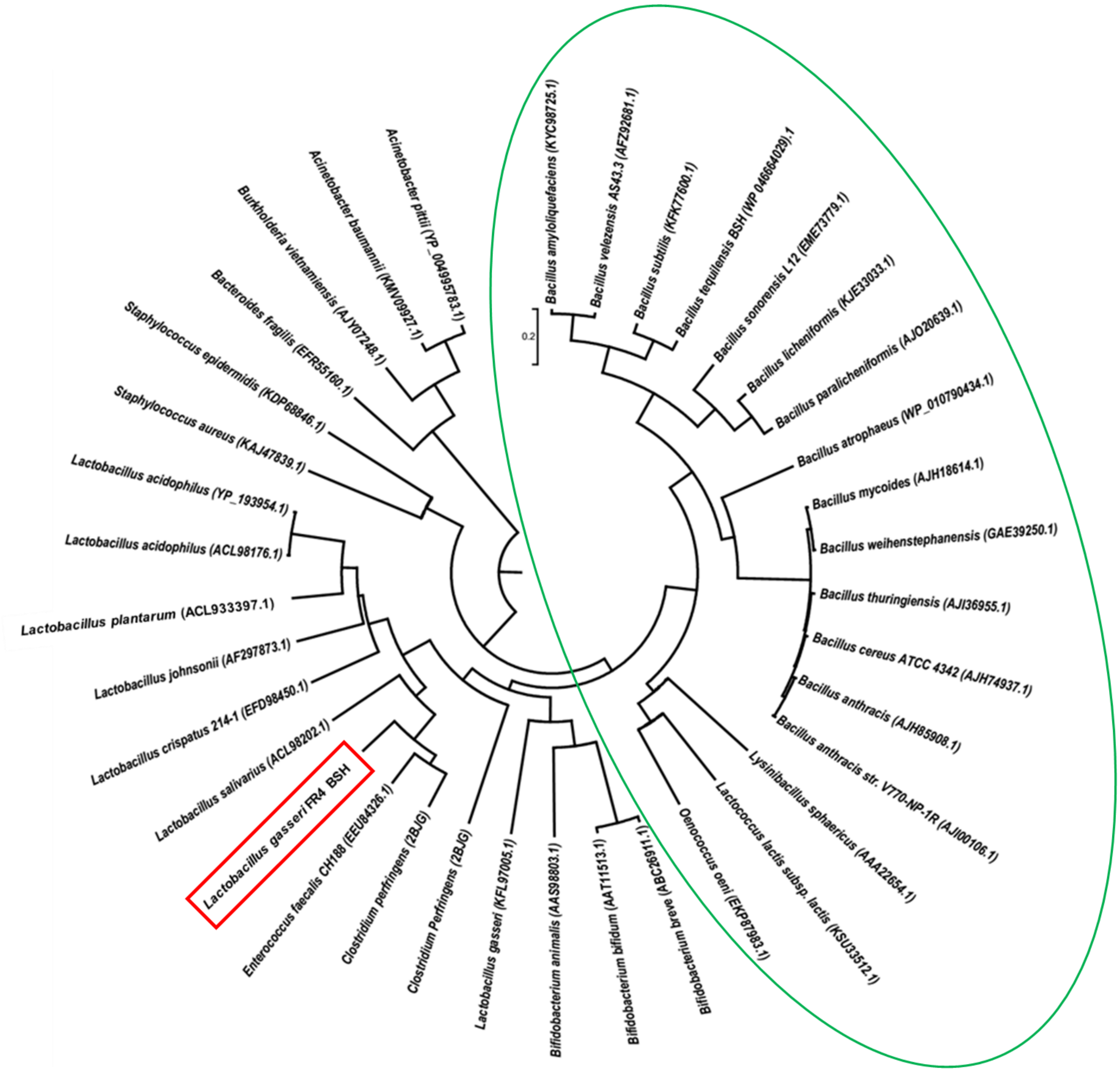

Fig. S4: Phylogenetic analysis of *Lg*BSH with BSH and PVA of various bacterial species was performed using UPGMA method. The PVA sequences were marked by green circle. The optimal tree with the sum of branch length = 9.23244762 is shown. The tree is drawn to scale, with branch lengths in the same units as those of the evolutionary distances used to infer the phylogenetic tree. The evolutionary distances were computed using the Poisson correction method and are in the units of the number of amino acid substitutions per site. The analysis involved 37 amino acid sequences. All positions containing gaps and missing data were eliminated. Evolutionary analyses were conducted in MEGA6 software.

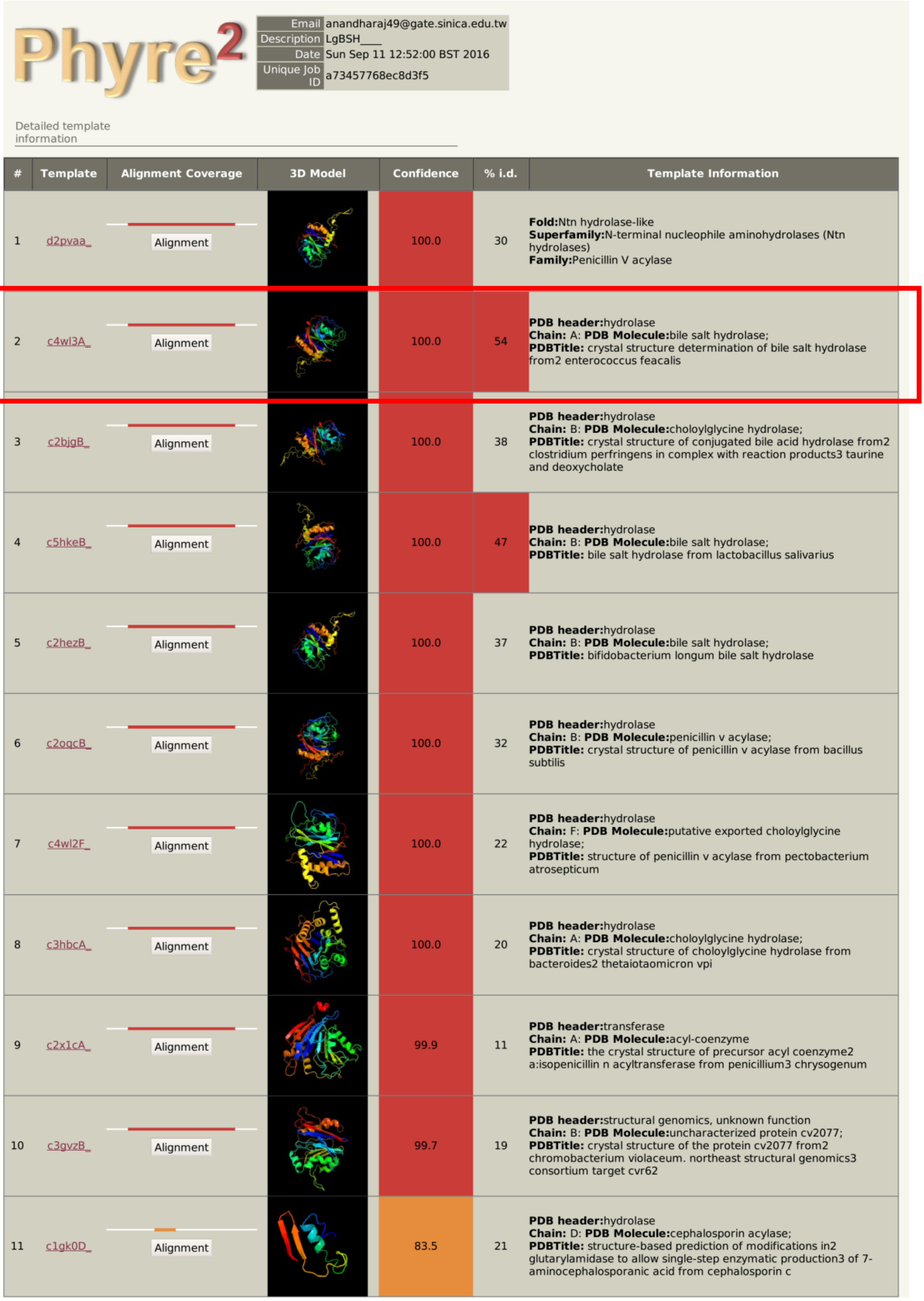

Phyre2

Email	anandharaj49@gate.sinica.edu.tw
Description	LgBSH___
Date	Sun Sep 11 12:52:00 BST 2016
Unique Job ID	a73457768ec8d3f5

Detailed template information

#	Template	Alignment Coverage	3D Model	Confidence	% i.d.	Template Information
1	d2pvaa_	Alignment		100.0	30	**Fold:**Ntn hydrolase-like **Superfamily:**N-terminal nucleophile aminohydrolases (Ntn hydrolases) **Family:**Penicillin V acylase
2	c4wl3A_	Alignment		100.0	54	**PDB header:**hydrolase **Chain:** A: **PDB Molecule:**bile salt hydrolase; **PDBTitle:** crystal structure determination of bile salt hydrolase from2 enterococcus feacalis
3	c2bjgB_	Alignment		100.0	38	**PDB header:**hydrolase **Chain:** B: **PDB Molecule:**choloylglycine hydrolase; **PDBTitle:** crystal structure of conjugated bile acid hydrolase from2 clostridium perfringens in complex with reaction products3 taurine and deoxycholate
4	c5hkeB_	Alignment		100.0	47	**PDB header:**hydrolase **Chain:** B: **PDB Molecule:**bile salt hydrolase; **PDBTitle:** bile salt hydrolase from lactobacillus salivarius
5	c2hezB_	Alignment		100.0	37	**PDB header:**hydrolase **Chain:** B: **PDB Molecule:**bile salt hydrolase; **PDBTitle:** bifidobacterium longum bile salt hydrolase
6	c2oqcB_	Alignment		100.0	32	**PDB header:**hydrolase **Chain:** B: **PDB Molecule:**penicillin v acylase; **PDBTitle:** crystal structure of penicillin v acylase from bacillus subtilis
7	c4wl2F_	Alignment		100.0	22	**PDB header:**hydrolase **Chain:** F: **PDB Molecule:**putative exported choloylglycine hydrolase; **PDBTitle:** structure of penicillin v acylase from pectobacterium atrosepticum
8	c3hbcA_	Alignment		100.0	20	**PDB header:**hydrolase **Chain:** A: **PDB Molecule:**choloylglycine hydrolase; **PDBTitle:** crystal structure of choloylglycine hydrolase from bacteroides2 thetaiotaomicron vpi
9	c2x1cA_	Alignment		99.9	11	**PDB header:**transferase **Chain:** A: **PDB Molecule:**acyl-coenzyme **PDBTitle:** the crystal structure of precursor acyl coenzyme2 a:isopenicillin n acyltransferase from penicillium3 chrysogenum
10	c3gvzB_	Alignment		99.7	19	**PDB header:**structural genomics, unknown function **Chain:** B: **PDB Molecule:**uncharacterized protein cv2077; **PDBTitle:** crystal structure of the protein cv2077 from2 chromobacterium violaceum. northeast structural genomics3 consortium target cvr62
11	c1gk0D_	Alignment		83.5	21	**PDB header:**hydrolase **Chain:** D: **PDB Molecule:**cephalosporin acylase; **PDBTitle:** structure-based prediction of modifications in2 glutarylamidase to allow single-step enzymatic production3 of 7-aminocephalosporanic acid from cephalosporin c

Fig. S5: Template selection using Phyre2 software. The BSH from *E. faecalis* (4WL3) was selected as a template for homology modeling.

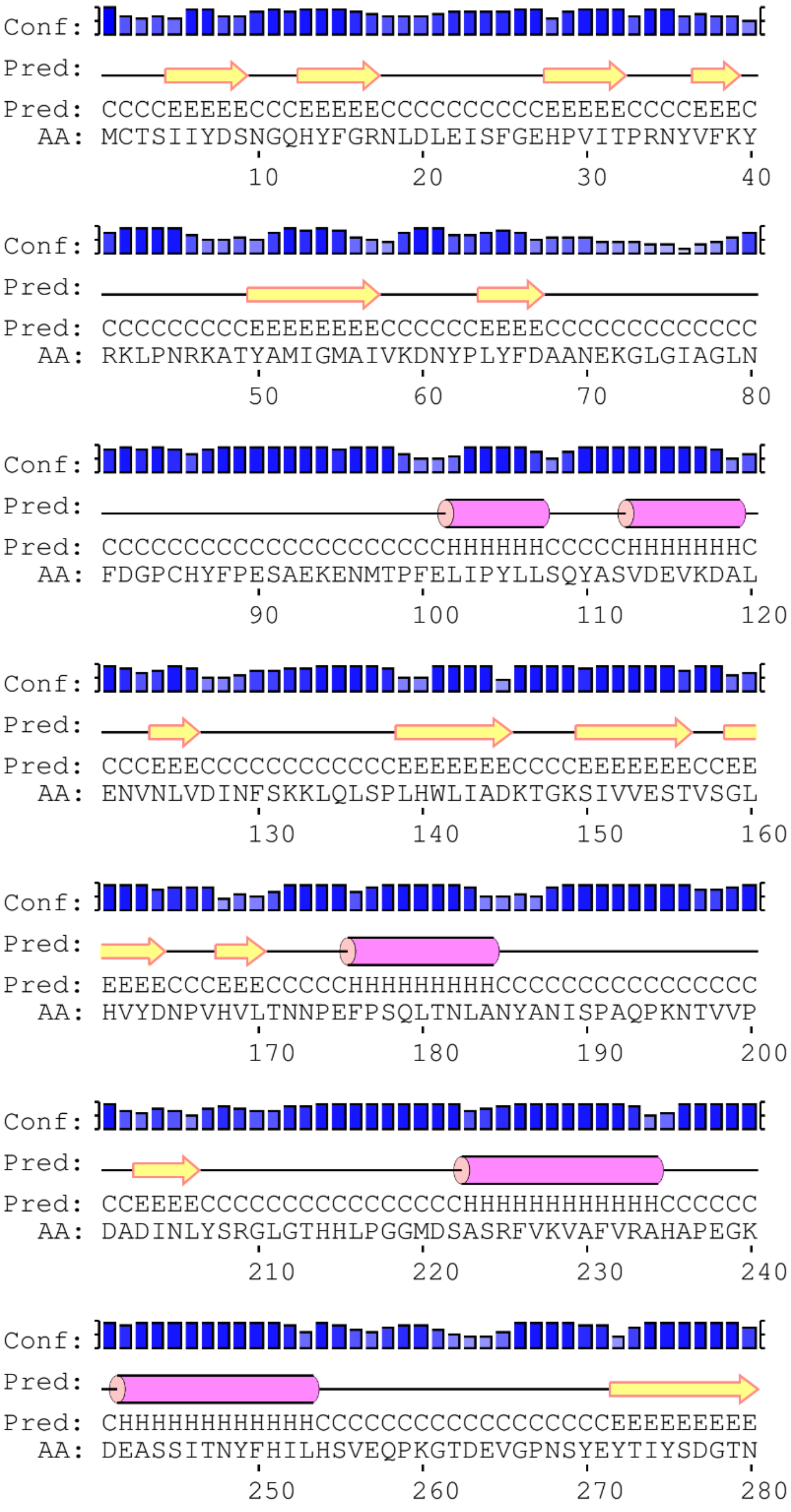
Conf:
Pred:
Pred: CCCCEEEEECCCEEEEECCCCCCCCCCEEEEECCCCEEEC
AA: MCTSIIYDSNGQHYFGRNLDLEISFGEHPVITPRNYVFKY
10 20 30 40
Conf:
Pred:
Pred: CCCCCCCCCEEEEEEEECCCCCCEEEECCCCCCCCCCCCC
AA: RKLPNRKATYAMIGMAIVKDNYPLYFDAANEKGLGIAGLN
50 60 70 80
Conf:
Pred:
Pred: CCCCCCCCCCCCCCCCCCCCCCHHHHHHCCCCHHHHHHHC
AA: FDGPCHYFPESAEKENMTPFELIPYLLSQYASVDEVKDAL
90 100 110 120
Conf:
Pred:
Pred: CCCEEECCCCCCCCCCCCEEEEEEECCCCEEEEEEECCEE
AA: ENVNLVDINFSKKLQLSPLHWLIADKTGKSIVVESTVSGL
130 140 150 160
Conf:
Pred:
Pred: EEEECCCEEECCCCCHHHHHHHHHCCCCCCCCCCCCCCCC
AA: HVYDNPVHVLTNNPEFPSQLTNLANYANISPAQPKNTVVP
170 180 190 200
Conf:
Pred:
Pred: CCEEEECCCCCCCCCCCCCCCCHHHHHHHHHHHHCCCCCC
AA: DADINLYSRGLGTHHLPGGMDSASRFVKVAFVRAHAPEGK
210 220 230 240
Conf:
Pred:
Pred: CHHHHHHHHHHHHCCCCCCCCCCCCCCCCCCEEEEEEEEE
AA: DEASSITNYFHILHSVEQPKGTDEVGPNSYEYTIYSDGTN
250 260 270 280

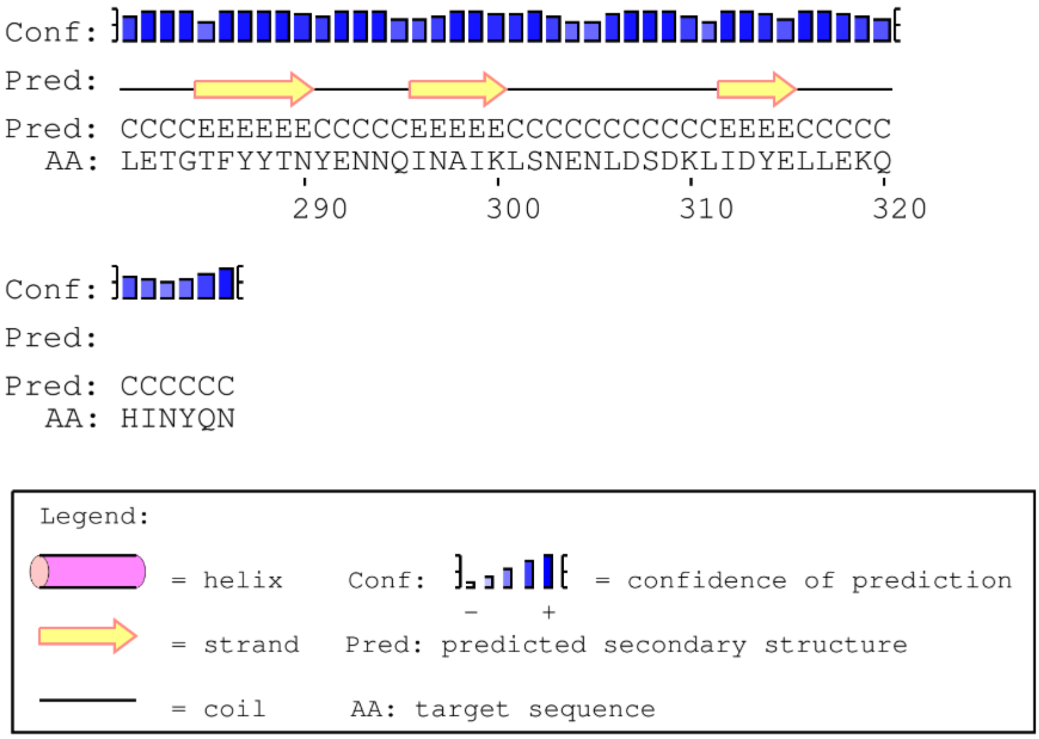

Fig. S6. Secondary structure prediction of *Lg*BSH using PSIPRED V3.3.

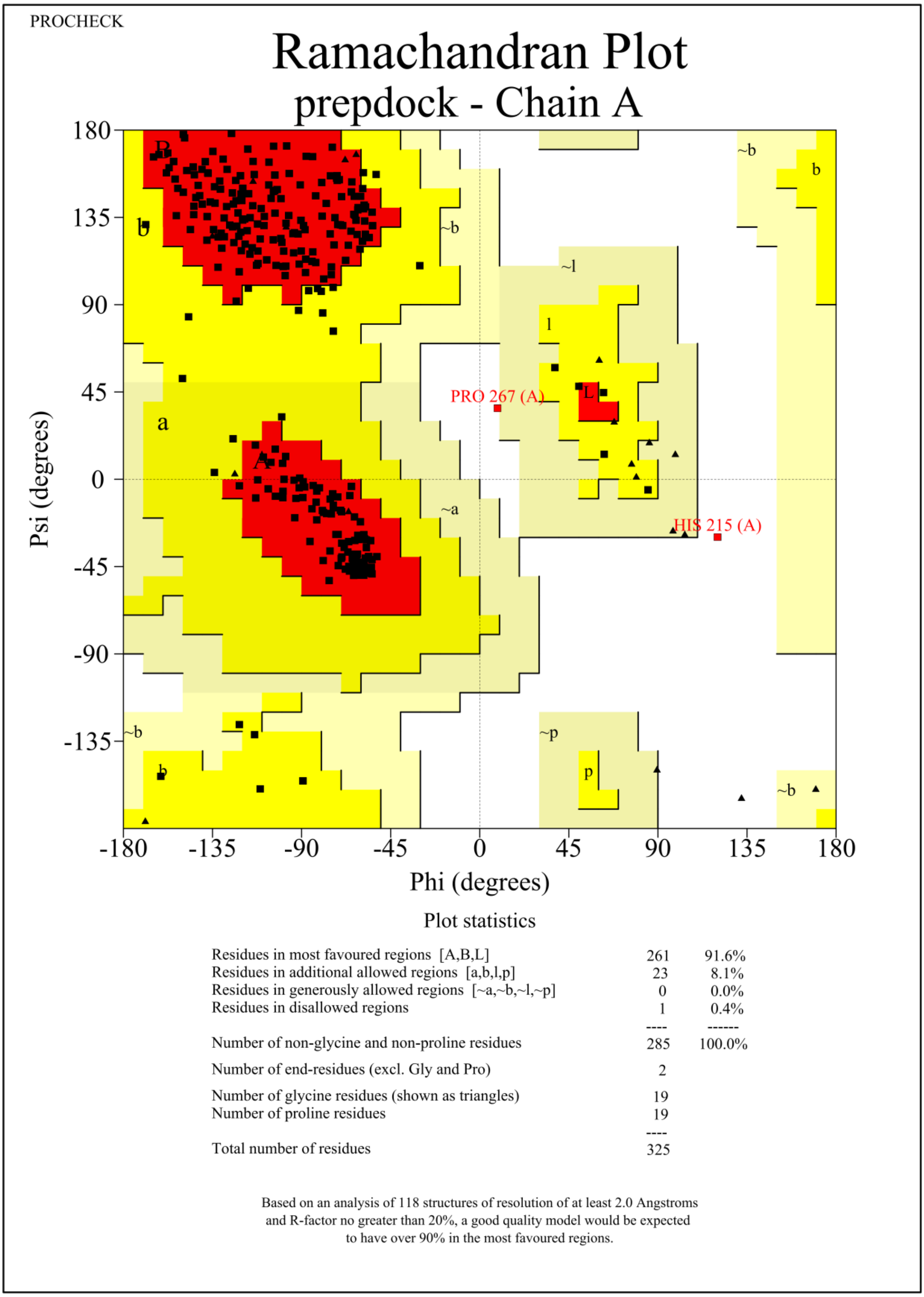

Fig. S7: Ramachandran plot analysis of *Lg*BSH using PSVS online software.

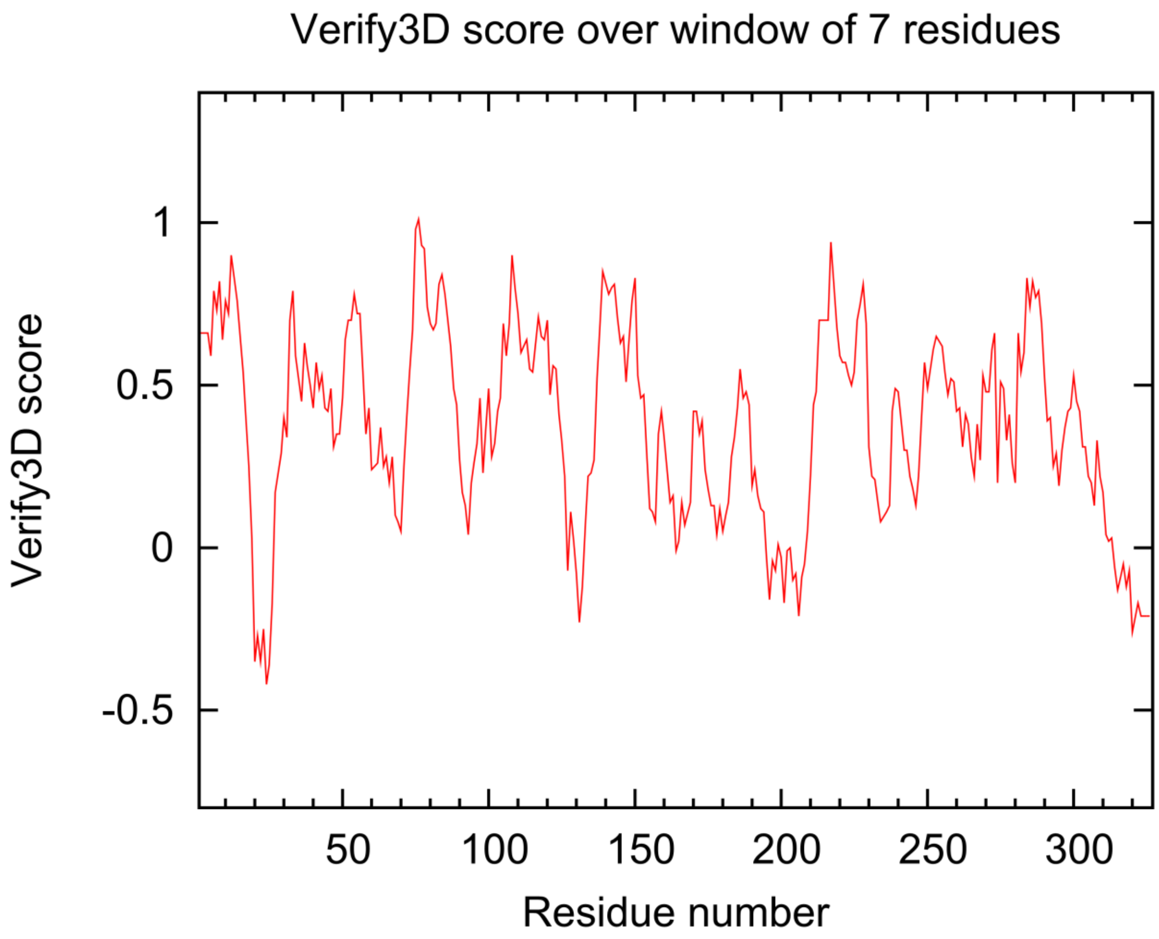

Fig. S8: The Verify 3D analysis of *Lg*BSH

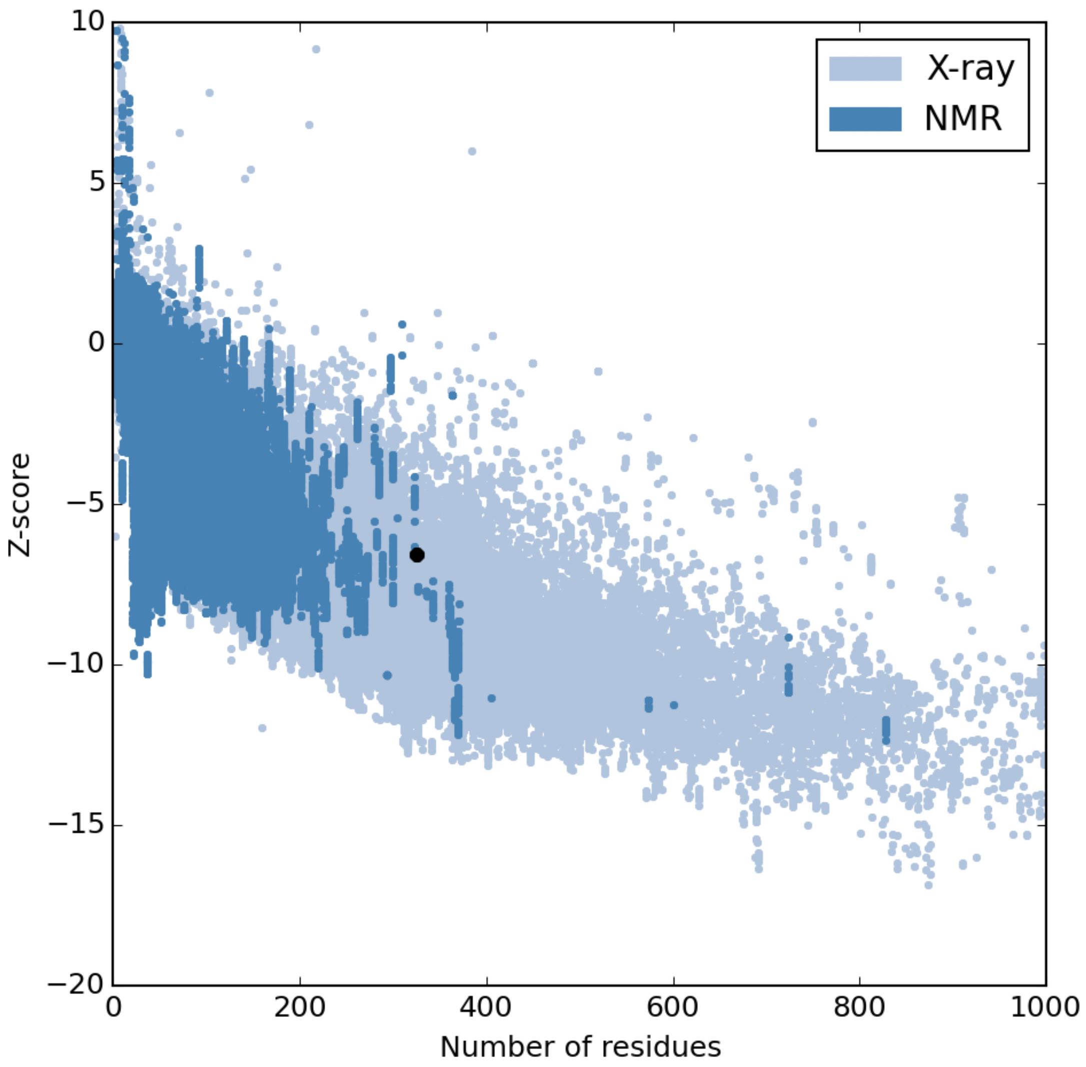

Fig. S9: The overall Z-score values of *Lg*BSH analyzed by using ProSA

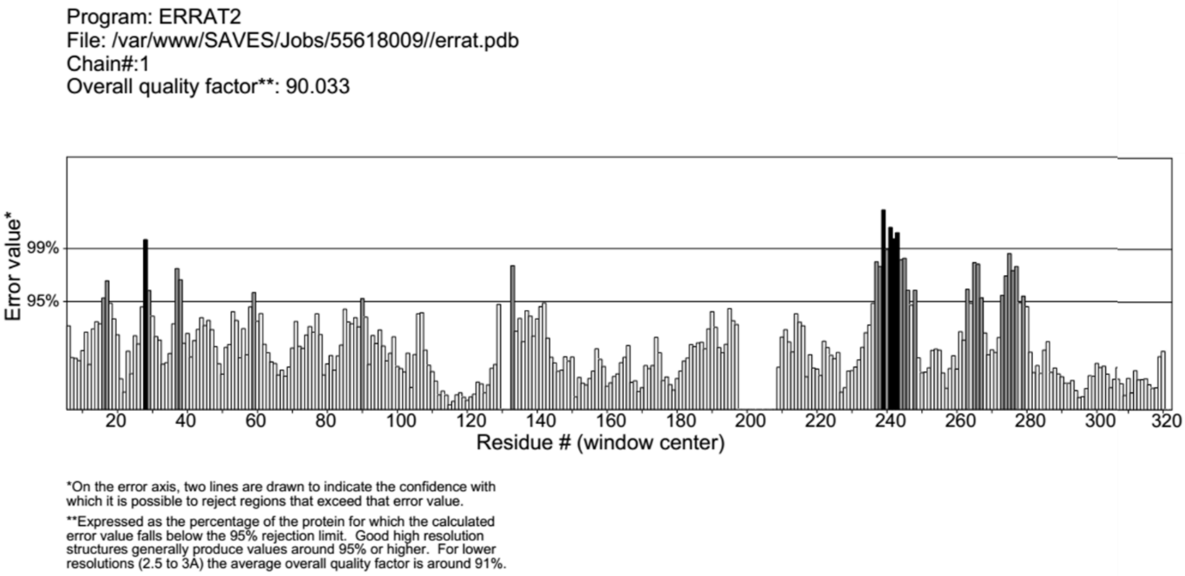

Fig. S10: Overall structural quality factor for *Lg*BSH evaluated by ERRAT web server.

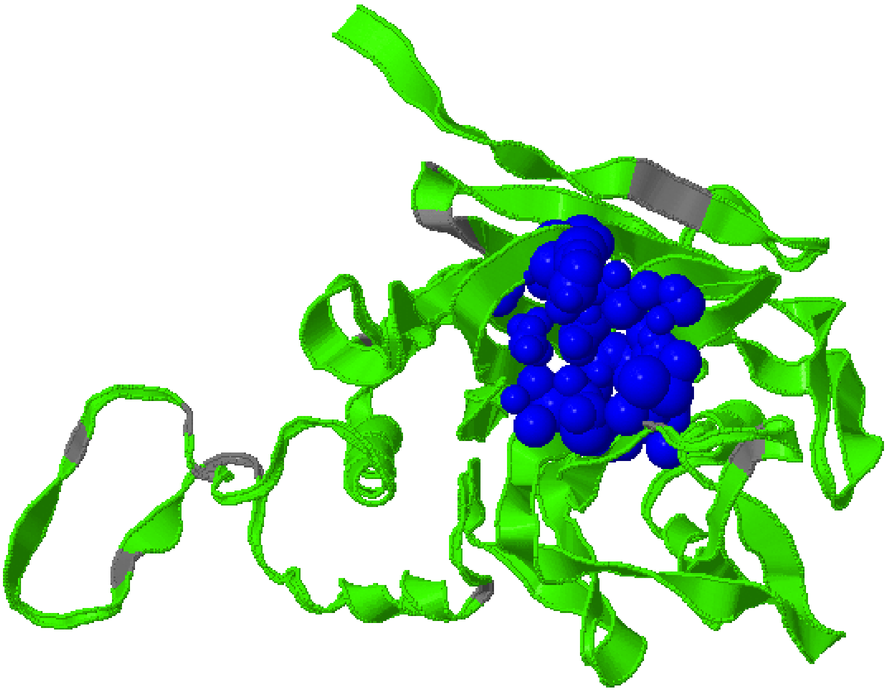

Fig. S11: Substrate binding pocket analysis of *Lg*BSH using CASTp online software. Residues involved in the substrate binding includes Cys2, Arg17, Leu19, Asp20, Leu21, Phe25, Lys59, Leu64, Tyr65, Phe66, Gly78, Asn80, Pro99, Phe100, Ile103, Pro104, Gly135, Ser137 and Leu139.
